# Supplementary material for: Neuromedin U and neurotensin may promote the development of the tumour microenvironment in neuroblastoma
Source: PeerJ. 2021 Jun 1;9:e11512. doi: 10.7717/peerj.11512 (PMC8176915; doi:10.7717/peerj.11512)
Supplement: Supplemental Information 1 [file peerj-09-11512-s001.docx]

**Supplementary table 1: Patients’ clinical characteristics in GSE73517 dataset**

| **Characteristics** |  | **Patients number** |
| --- | --- | --- |
| Age (year) | >=1.5 | 43 |
|  | <1.5 | 62 |
| INSS stage | 1 | 10 |
|  | 2 | 9 |
|  | 3 | 10 |
|  | 4 | 56 |
|  | 4S | 20 |
| Risk category | Low | 40 |
|  | Intermediate | 9 |
|  | High | 56 |
| MYCN status | Amplified | 33 |
|  | Non-amplified | 72 |
| 1p status | Deletion | 44 |
|  | Gain | 4 |
|  | Normal | 57 |
| 11q status | Deletion | 29 |
|  | Gain | 1 |
|  | Whole loss | 23 |
|  | Normal | 52 |
| 17q status | Gain | 92 |
|  | Normal | 13 |

**Supplementary table 1: Patients’ clinical characteristics in GSE120572 dataset**

| **Characteristics** |  | **Patients number** |
| --- | --- | --- |
| Age (year) | >=1.5 | 199 |
|  | <1.5 | 195 |
| INSS stage | 1 | 55 |
|  | 2 | 67 |
|  | 3 | 58 |
|  | 4 | 168 |
|  | 4S | 46 |
| MYCN status | Amplified | 83 |
|  | Non-amplified | 310 |
|  | N/A | 1 |

**Supplementary table 1: MYCN status in stage 4 and stage 4S**

|  |  | **Patients number** | |
| --- | --- | --- | --- |
| **INSS Satge** | **Status** | **GSE73517** | **GSE120572** |
| Stage 4 | Amplified | 26 | 58 |
|  | Non-amplified | 30 | 109 |
| Stage 4S | Amplified | 3 | 6 |
|  | Non-amplified | 17 | 40 |
