# Supplementary material for: Neuromedin U and neurotensin may promote the development of the tumour microenvironment in neuroblastoma
Source: PeerJ. 2021 Jun 1;9:e11512. doi: 10.7717/peerj.11512 (PMC8176915; doi:10.7717/peerj.11512)
Supplement: Supplemental Information 2 [file peerj-09-11512-s002.docx]

**Supplementary table 2: Clinical characteristics of neuroblastoma patients in TARGET database.**

| **Characteristics** |  | **Patients number** |
| --- | --- | --- |
| Gender | Female | 62 |
|  | Male | 88 |
| Age | >=1.5 | 118 |
|  | <1.5 | 32 |
| Race | Black or African American | 27 |
|  | Native Hawaiian or other Pacific Islander | 2 |
|  | White | 108 |
|  | Unknown | 13 |
| Status | Alive | 75 |
|  | Dead | 75 |
| INSS Stage | 1 | 0 |
|  | 2 | 1 |
|  | 3 | 9 |
|  | 4 | 120 |
|  | 4S | 20 |
| MYCN status | Amplified | 30 |
|  | Non-amplified | 119 |
|  | Unknown | 1 |
| Histology | Favorable | 31 |
|  | Unfavorable | 109 |
|  | Unknown | 10 |
| Grade | Differentiating | 9 |
|  | Undifferentiated or Poorly Differentiated | 118 |
|  | Unknown | 23 |
| COG Risk Group | High Risk | 119 |
|  | Intermediate Risk | 17 |
|  | Low Risk | 14 |

**Supplementary table 2: MYCN status in stage 4 and stage 4S**

| **INSS Satge** | **Status** | **Patients number** |
| --- | --- | --- |
| Stage 4 | Amplified | 27 |
|  | Non-amplified | 92 |
| Stage 4S | Amplified | 1 |
|  | Non-amplified | 19 |
