# Supplementary material for: Neuromedin U and neurotensin may promote the development of the tumour microenvironment in neuroblastoma
Source: PeerJ. 2021 Jun 1;9:e11512. doi: 10.7717/peerj.11512 (PMC8176915; doi:10.7717/peerj.11512)
Supplement: Supplemental Information 3 — Results of biological process enrichment analysis for differentially expressed genes, which were intersected from datasets GSE120572 and GSE73517. [file peerj-09-11512-s003.docx]

Supplementary Table 2. Results of biological process enrichment analysis for differentially expressed genes, which were intersected from datasets GSE120572 and GSE73517

| **geneSet** | **description** | **FDR** | **userId** |
| --- | --- | --- | --- |
| GO:0051094 | positive regulation of developmental process | 7.24E-04 | ADRB2;APOA1;APOB;BMP7;CBLN1;NMU;CD86;CITED2;CNR1;DDAH1;FGB;GATA6;GHR;GREM1;GRID2;HK2;HMGA2;IL7;INSM1;LPAR1;NLRP3;NR1H4;NRCAM;NTRK1;PLXNC1;POU4F2;PTPRD;RIMS1;SOX6;TERT;TWIST1;VSTM2A;WNT4;ZEB2;ZFPM2;NTS |
| GO:0048585 | negative regulation of response to stimulus | 0.001861 | ADRB2;AMBP;APOA1;APOH;ARG1;BMP7;CBLN1;CGNL1;CNR1;COL3A1;DACT2;DDAH1;EPHA5;EPHA7;NMU;EYA4;FGB;GREM1;GRID2;GSC;HMGA2;IGFBP1;IL7;LPAR1;NCOA7;NLRP3;NR1H4;NTRK1;PRAME;RGS9;SCG2;SPINK5;NTS;TCF21;TERT;TWIST1;WNT4 |
| GO:0035295 | tube development | 0.002515 | APOB;APOH;ARG1;BMP7;CALB1;CITED2;COL3A1;DACT2;DDAH1;EPHA7;EYA1;FAT4;GATA6;GREM1;GSC;HK2;HMGA2;NRCAM;SCG2;SGCD;SPINK5;TCF21;TERT;TWIST1;WNT4;ZEB2;ZFPM2 |
| GO:0007155 | cell adhesion | 0.004014 | AMBP;APOA1;NTS;BMP7;CBLN1;CD86;CDH12;CDH18;CDH19;CDH22;CITED2;COL3A1;DACT2;EPB41L4B;EPHA7;FAT4;FGB;GREM1;GRID2;IL7;LAMA3;MDGA;NCAN;NLRP3;NRCAM;PCDH7;PLXNC1;PRPH2;PTPRD;SPINK5;VSTM2L |
| GO:0022610 | biological adhesion | 0.004014 | AMBP;APOA1;ARG1;BMP7;CBLN1;CD86;CDH12;CDH18;CDH19;CDH22;CITED2;COL3A1;DACT2;EPB41L4B;EPHA7;FAT4;FGB;GREM1;GRID2;IL7;LAMA3;MDGA;NCAN;NLRP3;NRCAM;PCDH7;PLXNC1;PRPH2;PTPRD;SPINK5;VSTM2L;WNT4 |
| GO:2000026 | regulation of multicellular organismal development | 0.00495 | ADRB2;APOA1;APOH;BMP7;CAMK4;CBLN1;CD86;CITED2;CNR1;COL3A1;DDAH1;EPHA7;EYA1;FAT4;GATA6;GREM1;GRID2;NTS;HMGA2;IL7;LAMA3;LPAR1;MDGA1;NLRP3;NR1H4;NRCAM;NTRK1;PLXNC1;POU4F2;PTPRD;RGN;NMU;SOX6;SPINK5;TERT;TWIST1;WNT4;ZEB2;ZFPM2 |
| GO:0048699 | generation of neurons | 0.005895 | ADCY1;APOA1;BMP7;CBLN1;CNR1;COL3A1;CRB1;DNER;EPB41L3;EPHA5;EYA1;EPHA7;FAT4;FEV;GFRA3;GRID2;INSM1;IRX5;LPAR1;MDGA1;NRCAM;WNT4;NTRK1;PLXNC1;POU4F2;PTPRD;RIMS1;SPINK5;TERT;TWIST1;VSTM2L; ZEB2 |
| GO:0022603 | regulation of anatomical structure morphogenesis | 0.005895 | APOA1;APOH;BMP7;CITED2;DDAH1;EPB41L3;EPHA7;EYA1;FGB;GAS2;GATA6;GREM1;HK2;HMGA2;LPAR1;NRCAM;PLXNC1;POU4F2;PTPRD;RIMS1;SPINK5;TERT;TWIST1;WNT4;ZEB2 |
| GO:0035239 | tube morphogenesis | 0.005895 | APOB;APOH;BMP7;COL3A1;DDAH1;EPHA7;EYA1;FAT4;GATA6;GREM1;HK2;HMGA2;NRCAM;SCG2;SGCD;SPINK5;TCF21;TERT;TWIST1;WNT4;ZEB2;ZFPM2 |
| GO:0060548 | negative regulation of cell death | 0.005895 | ALB;APOH;BMP7;CITED2;EPHA5;EPHA7;EYA1;EYA4;FGB;GATA6;GREM1;HMGA2;IL7;NCOA7;NR1H4;NTRK1;PRAME;PRAMEF18;RGN;SCG2;TERT;TWIST1;VSTM2L;WNT4;ZFPM2;NMU |
| GO:0098609 | cell-cell adhesion | 0.005895 | APOA1;ARG1;BMP7;NTS;CD86;CDH12;CDH18;CDH19;CDH22;CITED2;EPHA7;FAT4;NMU;GRID2;IL7;MDGA1;NLRP3;NRCAM;PCDH7;PTPRD;VSTM2L;WNT4 |
| GO:0045597 | positive regulation of cell differentiation | 0.005895 | APOA1;APOB;BMP7;CD86;CNR1;NTS;GATA6;GREM1;IL7;INSM1;LPAR1;NLRP3;NRCAM;NTRK1;PLXNC1;POU4F2;PTPRD;RIMS1;SOX6;TERT;TWIST1;VSTM2A;WNT4;ZEB2;NMU; FGB |
| GO:0022008 | neurogenesis | 0.005895 | ADCY1;APOA1;BMP7;CBLN1;CNR1;COL3A1;CRB1;DNER;EPB41L3;EPHA5;EPHA7;EYA1;FAT4;FEV;GFRA3;GRID2;INSM1;IRX5;LPAR1;MDGA1;NRCAM;NTRK1;PLXNC1;POU4F2;PTPRD;RIMS1;SOX6;SPINK5;TERT;TWIST1;VSTM2L;WNT4;ZEB2 |
| GO:0048646 | anatomical structure formation involved in morphogenesis | 0.005895 | APOH;BMP7;CALB1;CBLN1;CITED2;DDAH1;EPB41L3;EXOC4;EYA1;GATA6;GREM1;GRID2;GSC;HK2;HMGA2;KRT19;LAMA3;MYH11;NRCAM;SCG2;SPINK5;TCF21;TERT;TWIST1;WNT4;ZEB2 |
| GO:0009968 | negative regulation of signal transduction | 0.005895 | ADRB2;AMBP;APOA1;ARG1;BMP7;CBLN1;CGNL1;DACT2;EPHA5;EPHA7;EYA1;EYA4;FGB;GREM1;GSC;IGFBP1;IL7;LPAR1;NLRP3;NR1H4;NTRK1;PRAME;RGS9;SCG2;TCF21;TERT;TWIST1;WNT4 |
