# Supplementary material for: Neuromedin U and neurotensin may promote the development of the tumour microenvironment in neuroblastoma
Source: PeerJ. 2021 Jun 1;9:e11512. doi: 10.7717/peerj.11512 (PMC8176915; doi:10.7717/peerj.11512)
Supplement: Supplemental Information 4 — 25 hub genes screened by CytoHubba ranked by Degree method. [file peerj-09-11512-s004.docx]

Supplementary Table 3. 25 hub genes screened by CytoHubba ranked by Degree method

| Rank | Name | Score |
| --- | --- | --- |
| 1 | APOA1 | 14 |
| 1 | ALB | 14 |
| 3 | APOB | 13 |
| 4 | SERPINC1 | 12 |
| 4 | ITIH2 | 12 |
| 6 | TF | 10 |
| 6 | AMBP | 10 |
| 8 | FGB | 9 |
| 8 | IGFBP1 | 9 |
| 10 | APOH | 8 |
| 10 | CYR61 | 8 |
| 10 | SCG2 | 8 |
| 13 | ADCY1 | 7 |
| 14 | NMU | 6 |
| 14 | LPAR1 | 6 |
| 14 | GC | 6 |
| 17 | ADRB2 | 5 |
| 18 | NTS | 4 |
| 18 | HRH1 | 4 |
| 18 | TTR | 4 |
| 18 | QRFPR | 4 |
| 22 | NR1H4 | 3 |
| 22 | CNR1 | 3 |
| 22 | PTH1R | 3 |
| 22 | CALCB | 3 |
