# Supplementary figures and images for: Neuromedin U and neurotensin may promote the development of the tumour microenvironment in neuroblastoma
Source: PeerJ. 2021 Jun 1;9:e11512. doi: 10.7717/peerj.11512 (PMC8176915; doi:10.7717/peerj.11512)

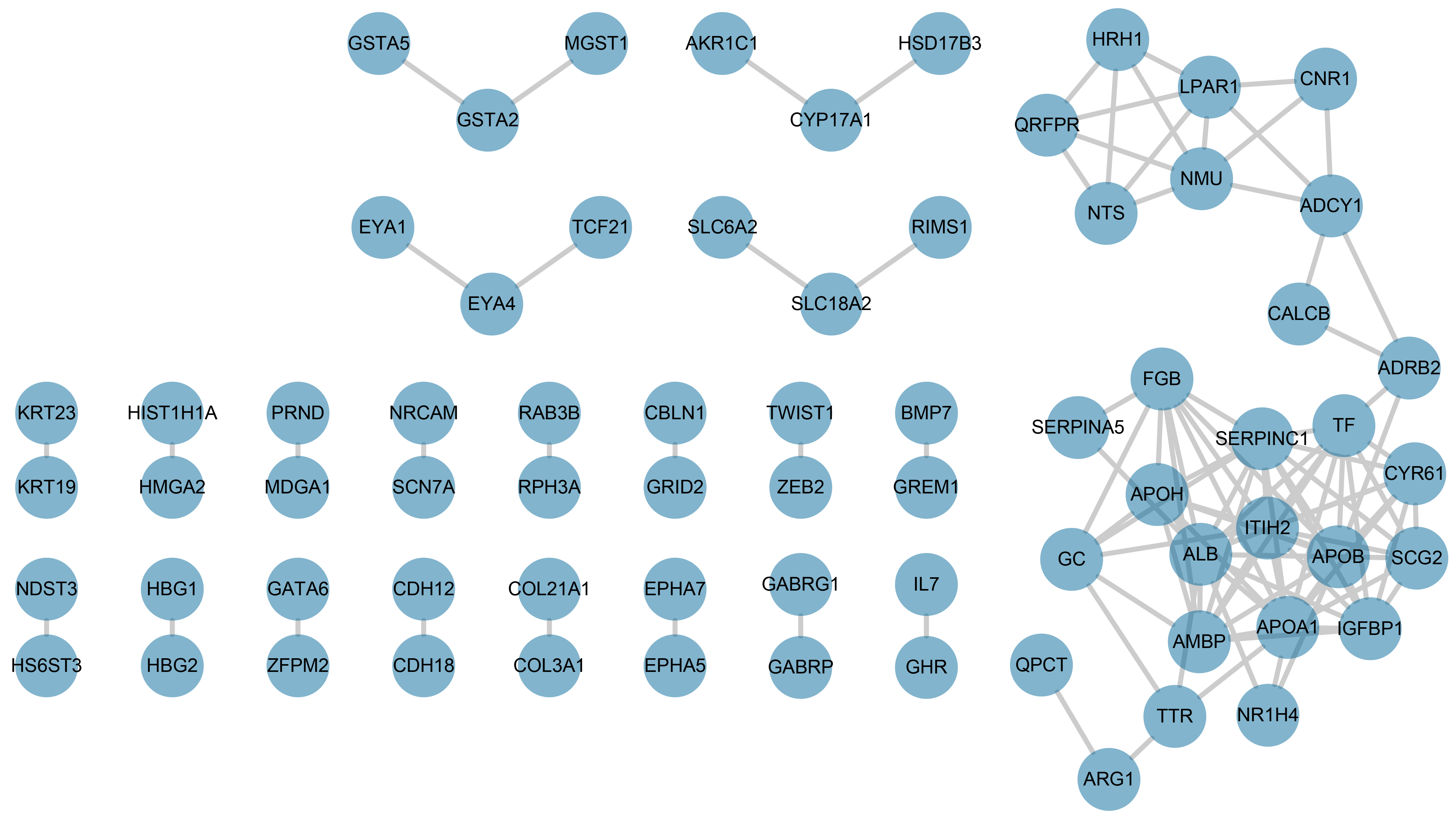

Supplement: Supplemental Information 5 — The protein to protein interaction analysis of 216 intersected genes. [file peerj-09-11512-s005.png]

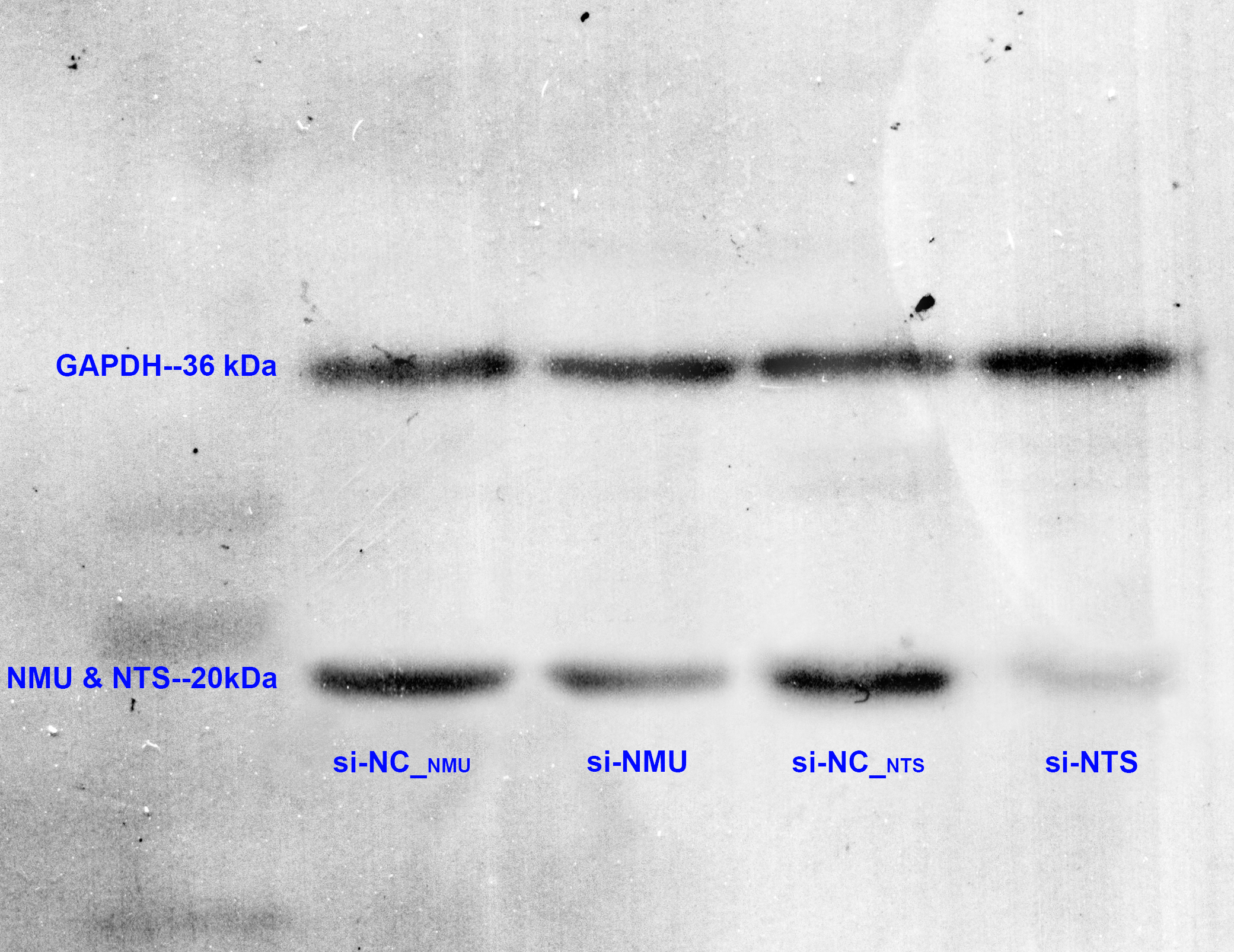

Supplement: Supplemental Information 6 — The protein as internal reference for western blot assay was GAPDH, which the predicted molecular weight is 36 kDa, and the predicted molecular weight of NMU and NTS are both 20 kDa. [file peerj-09-11512-s006.zip › Western blot for Figure 4/Western blot for Figure 4.jpg]
