# Supplementary material for: Neuromedin U and neurotensin may promote the development of the tumour microenvironment in neuroblastoma
Source: PeerJ. 2021 Jun 1;9:e11512. doi: 10.7717/peerj.11512 (PMC8176915; doi:10.7717/peerj.11512)
Supplement: Supplemental Information 7 — Raw data of invaded cell numbers by interfering expression of NMU and NTS, respectively. [file peerj-09-11512-s007.docx]

**NMU:** **Invased cell numbers**

| **si-NMU** | **si-NC** |
| --- | --- |
| 607 | 1245 |
| 635 | 1340 |
| 601 | 1059 |
| 597 | 1158 |
| 665 | 1258 |
| 539 | 1224 |
| 582 | 1410 |
| 619 | 1033 |
| 602 | 991 |
| 711 | 1201 |

**NTS: Invased cell numbers**

| **si-NTS** | **si-NC** |
| --- | --- |
| 741 | 1490 |
| 699 | 1091 |
| 596 | 1439 |
| 878 | 1523 |
| 701 | 1129 |
| 732 | 1287 |
| 710 | 983 |
| 682 | 1288 |
| 809 | 1297 |
| 859 | 1451 |
